# What More Can Be Delivered to Future Patients with Coronary Syndromes?

**DOI:** 10.3390/jcm11195704

**Published:** 2022-09-27

**Authors:** Atsushi Tanaka, Koichi Node

**Affiliations:** Department of Cardiovascular Medicine, Saga University, Saga 849-8501, Japan

Coronary artery disease (CAD) is a major cardiovascular disease that imposes substantial clinical and socioeconomic burdens worldwide. To date, many studies have been conducted to address this issue, leading to innovations and advances in various medical technologies and treatments. Recently, based on its dynamic nature in clinical settings, the clinical concept of CAD has been subdivided into “acute coronary syndrome (ACS)” and “chronic coronary syndrome (CCS)” [1]. In this context, invasive and multidisciplinary therapies in the acute setting have been the mainstay of ACS treatment and have significantly contributed to improving patient survival rates. Simultaneously, the appropriate practice of optimal drug therapy has been recommended in relevant guidelines for the chronic management of CCS, which has contributed to the prevention of CAD recurrence and improved prognosis. However, CAD remains a problem worldwide, and the reality is that many patients still develop CAD. This raises the question: what is needed in the future to further overcome CAD? We aimed to answer this question in this study.

To begin with, we believe that there is a strong need to accumulate novel scientific and clinical knowledge in this field to develop seamless treatment and preventive strategies for acute and chronic coronary syndrome. Furthermore, accumulated data indicate that there are some residual risk factors for coronary syndrome combined with ACS and CCS. Moreover, the clinical management of cardiovascular/non-cardiovascular complications associated with coronary syndrome to achieve better outcomes is a critical issue. Therefore, we need to gather potential papers to solve these unmet needs in the clinical management of coronary syndrome in contemporary medical care settings. With the indispensable help of excellent authors and reviewers, our group was able to collate 13 papers on this topic that were published in the past two years. In this editorial, we would like to express our gratitude to those contributors and provide a very concise introduction to each of these papers (please refer to each publication for details).

Two papers on ischemia imaging have been published by Japanese researchers. Kamo et al. [2] reported that the subtraction computed tomography (CT) fractional flow reserve method was useful for improving the diagnostic performance of hemodynamically significant coronary stenosis in patients with severe calcification. Kouchi et al. [3] found that the myocardial perfusion ratio to the aorta derived from myocardial CT perfusion analysis had a higher diagnostic accuracy for detecting impaired myocardial perfusion than conventional semi-quantitative parameters, such as myocardial CT attenuation and transmural perfusion ratio.

Two papers on percutaneous coronary intervention (PCI) were published in Korea and Japan. Kim et al. [4] revealed that differences in reperfusion strategies did not significantly affect prognosis in patients with multivessel non-ST-elevation myocardial infarction and chronic kidney disease, emphasizing the need for further investigation of the optimal perfusion strategy. Noma et al. [5] reported that adjunctive catheter-derived thrombolysis with intracoronary monteplase delivery resulted in favorable outcomes in patients with ST-elevation myocardial infarction, especially in those with a high thrombus burden who were refractory to primary PCI procedures.

Two studies on coronary spasm were conducted in Japan and Korea. Teragawa et al. [6] identified active smoking as a possible risk factor associated with the increase in the index of microcirculatory resistance (IMR) for patients who underwent an invasive microvascular vasodilatory function test, showing that IMR differed according to the coronary artery branches and types of coronary spasm. Kim et al. [7] documented that the chronic use of nitrates in patients with vasospastic angina, relative to other types of vasodilators, such as nicorandil, was associated with an increased risk of adverse clinical outcomes in a prospective nationwide registry database.

Three studies on pharmacotherapy were also conducted in Korea and Japan. Kim et al. [8] reported that the prognostic benefit of statin therapy following PCI for acute myocardial infarction (AMI) was significant even in patients with advanced renal impairment, compared to non-statin users. However, since the incidence rate of adverse events in patients with chronic kidney disease is still high, further therapeutic approaches are needed, especially in these patient populations. Jo et al. [9] performed a multicenter, double-blind, randomized trial and found that a single pill of olmesartan/amlodipine plus rosuvastatin therapy was more effective and safe for the management of both hypertension and dyslipidemia than either olmesartan plus rosuvastatin or olmesartan plus amlodipine therapy. Mori et al. [10] further provided real-world evidence for the use of a modified Japanese dose of prasugrel (loading/maintenance: 20/3.75 mg) with a similar efficacy and safety to the standard dose of clopidogrel in patients with AMI.

Three papers investigating uric acid as a residual risk factor for coronary syndrome were published by Japanese and Italian researchers. Hiraga et al. demonstrated that serum uric acid (SUA) level was an independent factor inversely associated with systemic endothelial function in patients with a broad spectrum of CAD, including ischemia with non-obstructive CAD [11]. Watanabe et al. found that plasma xanthine oxidoreductase activity was an independent predictor of coronary spasm in both sexes and that this effect was especially pronounced in female patients [12]. Maloberti et al. [13] reviewed recent evidence to clarify the role of SUA in the care of coronary syndrome and concluded that further studies are warranted to determine the pathological and therapeutic relationship between SUA and CAD.

Finally, Goriki et al. [14] sought to create a laboratory-only risk score model to predict post-discharge mortality in survivors following AMI, revealing that a model comprising hemoglobin, renal function, albumin, and troponin I levels obtained prior to primary PCI could be clinically useful for the early risk stratification of one-year mortality.

However, patients are still developing coronary syndromes. We hope that what we have elucidated in this Topical Collection will help to better manage future patients with these diseases; however, we also understand that significant research is still required to overcome coronary syndromes. It is also our strong hope that this collection will promote further research to enable a better understanding and clinical management of coronary syndromes in the near future. Once again, we would like to express our gratitude to the authors and reviewers for their dedicated contributions to our Topical Collection “Coronary Syndrome: Clinical Treatment, Prevention and Management for Better Outcomes” in *the Journal of Clinical Medicine*.
